# Human microglia and astrocytes constitutively express the neurokinin-1 receptor and functionally respond to substance P

**DOI:** 10.1186/s12974-017-1012-5

**Published:** 2017-12-13

**Authors:** Amanda R. Burmeister, M. Brittany Johnson, Vinita S. Chauhan, Megan J. Moerdyk-Schauwecker, Ada D. Young, Ian D. Cooley, Alejandra N. Martinez, Geeta Ramesh, Mario T. Philipp, Ian Marriott

**Affiliations:** 1 Department of Biological Sciences, University of North Carolina at Charlotte, 9201 University City Blvd, Charlotte, NC 28223 USA; 2 Division of Bacteriology and Parasitology, Tulane National Primate Research Center, Covington, LA USA

**Keywords:** Substance P, Neurokinin-1 receptor, Neuroinflammation, Human, Microglia, Astrocyte

## Abstract

**Background:**

The tachykinin substance P (SP) is recognized to exacerbate inflammation at peripheral sites via its target receptor, neurokinin 1 receptor (NK-1R), expressed by leukocytes. More recently, SP/NK-1R interactions have been associated with severe neuroinflammation and neuronal damage. We have previously demonstrated that NK-1R antagonists can limit neuroinflammatory damage in a mouse model of bacterial meningitis. Furthermore, we have since shown that these agents can attenuate bacteria-induced neuronal and glial inflammatory mediator production in nonhuman primate (NHP) brain explants and isolated neuronal cells, and following in vivo infection.

**Methods:**

In the present study, we have assessed the ability of NHP brain explants, primary human microglia and astrocytes, and immortalized human glial cell lines to express NK-1R isoforms. We have utilized RT-PCR, immunoblot analysis, immunofluorescent microscopy, and/or flow cytometric analysis, to quantify NK-1R expression in each, at rest, or following bacterial challenge. Furthermore, we have assessed the ability of human microglia to respond to SP by immunoblot analysis of NF-kB nuclear translocation and determined the ability of this neuropeptide to augment inflammatory cytokine release and neurotoxic mediator production by human astrocytes using an ELISA and a neuronal cell toxicity assay, respectively.

**Results:**

We demonstrate that human microglial and astrocytic cells as well as NHP brain tissue constitutively express robust levels of the full-length NK-1R isoform. In addition, we demonstrate that the expression of NK-1R by human astrocytes can be further elevated following exposure to disparate bacterial pathogens or their components. Importantly, we have demonstrated that NK-1R is functional in both human microglia and astrocytes and show that SP can augment the inflammatory and/or neurotoxic immune responses of glial cells to disparate and clinically relevant bacterial pathogens.

**Conclusions:**

The robust constitutive and functional expression of the full-length NK-1R isoform by human microglia and astrocytes, and the ability of SP to augment inflammatory signaling pathways and mediator production by these cells, support the contention that SP/NK-1R interactions play a significant role in the damaging neuroinflammation associated with conditions such as bacterial meningitis.

## Background

The neuropeptide substance P (SP) and its selective receptor, the neurokinin-1 receptor (NK-1R), is expressed at high levels within the central nervous system (CNS) (as reviewed in [1, 2]). In addition to its functions as a neurotransmitter in the perception of pain and its essential role in gut motility, this tachykinin is now recognized to exacerbate inflammation at peripheral sites including the skin, lung, and gastrointestinal and urogenital tracts. Indeed, this neuropeptide appears to contribute to disease pathology for some infectious agents. For example, SP increases the bronchoconstriction and damaging cardiac inflammation following infection with respiratory syncytial virus and encephalomyocarditis virus, respectively [3, 4]. Likewise, SP contributes to the severity of inflammation associated with *Trypanosoma brucei brucei* infection and inflammation and granuloma size in a mouse model of *Taenia solium* cysticercosis [5–7].

Recently, a number of studies have identified a similar role for SP and NK-1R interactions in neuroinflammation (as discussed in [1, 2]), and our data suggests that SP exacerbates damaging inflammation within the CNS in animal models in response to disparate bacterial pathogens. We determined that the absence of SP/NK-1R interactions in SP receptor-deficient mice or prophylactic pharmacological NK-1R inhibition in wild type animals significantly reduces bacteria-induced neuroinflammation and resultant CNS damage [8, 9]. NK-1R null mice and mice treated with an NK-1R antagonist showed reduced inflammatory and maintained immunosuppressive cytokine production, as well as decreased astrogliosis, cellularity, and demyelination following intracerebral administration of the Gram-negative bacterial pathogens *Neisseria meningiditis* and *Borrelia burgdorferi*, or the Gram-positive bacterium *Streptococcus pneumoniae* [8, 9]. More recently, we have demonstrated that the specific NK-1R antagonist, aprepitant, limits inflammatory nervous system immune responses in a nonhuman primate (NHP) model of Lyme neuroborreliosis [10]. These animal studies therefore indicate that SP/NK-1R interactions are essential for the progression of damaging inflammation following bacterial CNS infection and raise the intriguing possibility that targeting the NK-1R could be useful as an adjunctive therapy for such conditions.

We have previously demonstrated that murine glial cells functionally express the NK-1R [11]. Importantly, we have shown that SP can exacerbate the inflammatory responses of both murine microglia and astrocytes to *N. meningiditis* and *B. burgdorferi* [9]. In the present study, we report that primary human glia and immortalized human glial cell lines, as well as NHP brain tissue, constitutively express robust levels of full-length NK-1R. Furthermore, we show that SP can augment the inflammatory and/or neurotoxic responses of human microglia and astrocytes to disparate and clinically relevant bacterial pathogens. Taken together, these results are consistent with our animal model studies and indicate that SP/NK-1R interactions could play a significant role in the initiation and/or progression of damaging inflammation in humans following bacterial CNS infection.

## Methods

### Bacterial propagation

First passage *B. burgdorferi* strain B31 clone 5A19 spirochetes, isolated from an ear biopsy of a previously infected mouse, were grown in Barbour-Stoenner-Kelly-H medium supplemented with 6% rabbit serum and antibiotics (rifampicin at 45.4 μg/mL, phosphomycin at 193 μg/mL, and amphotericin at 0.25 μg/mL; Sigma-Aldrich, St. Louis, MO) to late logarithmic phase under microaerophilic conditions. An inoculum containing 1 × 10^7^ spirochetes/mL in RPMI 1640 medium (Invitrogen, USA) was prepared for use in in vitro studies and to infect ex vivo NHP brain tissue as previously described [12]. For in vitro human glia infection studies, *Neisseria meningitidis* strain MC58 was cultured in Columbia broth on an orbital shaker at 37 °C with 5% CO_2_ [9]. *Streptococcus pneumoniae* strain CDC CS109, an isolate from a patient with meningitis, was grown from frozen stock on tryptic soy agar with 5% defibrinated sheep blood and subsequently cultured in Todd-Hewitt broth at 37 °C as previously described by our laboratory [8]. *Staphylococcus aureus* strain UAMS-1 was grown in lysogeny broth (LB) on an orbital shaker at 37 °C with 5% CO_2_ overnight.

### Nonhuman primate frontal cortex brain slice isolation and ex vivo infection

Freshly harvested frontal cortex tissues were collected at necropsy from four rhesus macaques (*Macaca mulatta*) that were scheduled for euthanasia due to chronic idiopathic diarrhea or had undergone trauma. Animals were euthanized in accordance with the recommendations of the American Veterinary Medical Association’s Panel on Euthanasia. The frontal cortex was sliced into 2-mm sections, and each section was placed in separate wells of 12-well plates. Each well contained 2 mL of RPMI 1640 medium (BioWhittaker, Walkersville, MD) supplemented with 10% FBS, as previously described [13]. Tissue sections were exposed to medium alone or to medium containing *B. burgdorferi* (1 × 10^7^ bacteria/mL) and were processed for analysis at the indicated time points.

### Source and propagation of human glial cell lines and primary cells

U87-MG, an immortalized human astrocytic cell line, was obtained from the ATCC (HTB-14). Cells were maintained in Eagle’s Minimum Essential Medium supplemented with 10% FBS and penicillin/streptomycin. The human microglial cell line, hμglia, was a kind gift from Dr. Jonathan Karn (Case Western Reserve University). These cells were derived from primary human cells transformed with lentiviral vectors expressing SV40 T antigen and hTERT and have been classified as microglia due to their microglia-like morphology; migratory and phagocytic activities; presence of the microglial cell surface markers CD11b, TGFβR, and P2RY12; and characteristic microglial RNA expression profile [14]. This cell line was maintained in Dulbecco’s modified Eagle medium supplemented with 5% FBS and penicillin/streptomycin. Primary human astrocytes and microglia were purchased from ScienCell Research Laboratories (Carlsbad, CA) and were cultured in medium supplied by the vendor.

### In vitro infection of human microglia and astrocytes and exposure to bacterial components

Cells (1.5 × 10^5^) seeded in 12-well flat-bottom tissue culture plates were infected with bacteria at the indicated multiplicities of infection (MOI) in antibiotic-free culture medium for 2 h prior to washing and addition of complete culture medium. Alternatively, human glial cells were exposed to Pam3Cys, polyinosinic:polycytidylic acid (poly I:C) sodium salt, bacterial lipopolysaccharide (LPS), and/or flagellin, ligands for TLR2, TLR3, TLR4, and TLR5, respectively. Pam3Cys was purchased from InvivoGen (San Diego, CA). Flagellin (isolated from *Salmonella typhimurium* strain 14028) was purchased from Enzolife sciences (Farmingdale, NY). LPS (isolate from *Escherichia coli*) and poly I:C were purchased from Sigma-Aldrich (St. Louis, MO). Following infection or exposure to bacterial products, cells were then cultured in the presence or absence of SP (Sigma-Aldrich) at a concentration of 5 or 10 nM. At the indicated time points, whole-cell protein isolates were collected and RNA was isolated for immunoblot analysis and semi-quantitative RT-PCR, respectively.

### RNA extraction and semi-quantitative reverse transcription PCR

Total RNA was isolated from cultured glial cells using Trizol Reagent (Thermo Fisher Scientific) according to the manufacturer’s instructions and quantified using a Nanodrop ND-1000 spectrophotometer. Prior to reverse transcription, RNA was treated with amplification grade DNase (Sigma-Aldrich) to remove genomic DNA. All RNA samples were diluted to the same concentration and reverse transcribed in the presence of random hexamers using 200 U of RNase H minus Moloney leukemia virus reverse transcriptase (Promega, Madison, WI) in the buffer supplied by the manufacturer. Semi-quantitative RT-PCR was performed on 5% of the reverse-transcribed cDNA product to assess the relative levels of expression of mRNA encoding NK-1R and the housekeeping gene product glyceraldehyde 3-phosphate dehydrogenase (GAPDH). Positive and negative strand PCR primers used, respectively, were AACCCAAGTTCGAACCAG and ATGTACCTATCAAAGGCCACAGCC to amplify mRNA encoding total NK-1R, TCTTCTTCCTCCTGCCCTACATC and AGCACCGGAAGGCATGCTTGAAGCCCA to amplify mRNA encoding full-length NK-1R, ATCCTGGTGGCGTTGGCAGTC and GAGAGATCTGGCCATGTCCATAAAGA to amplify mRNA encoding preprotachykinin (PPT), and CCATCACCATCTTCCAGGAGCGAG and CACAGTCTTCTGGGTGGCAGTGAT to amplify mRNA encoding GAPDH.

### Immunoblot analysis

Homogenates from NHP frontal cortical tissue and whole-cell protein isolates from human cell cultures were subjected to immunoblot analysis as we have previously described [11, 15] using a mouse monoclonal antibody directed against human NK-1R (ThermoFisher Scientific; clone ZN003). In some experiments, nuclear protein extracts were obtained from hμglia cells as follows. Cells were suspended in a pH 7.9 lysis buffer containing 10 mM HEPES, 1.5 mM MgCl_2_, 10 mM KCl, 0.5 mM DTT, 0.05% NP40, and protease inhibitor cocktail for 10 min at 4 °C. The nuclei and other fragments were pelleted by centrifugation and supernatants were retained as cytoplasmic fractions. Nuclei were lysed by exposure to pH 7.9 high salt buffer containing 5 mM HEPES, 1.5 mM MgCl_2_, 0.2 mM EDTA, 0.5 mM DTT, 26% glycerol, and 300 mM NaCl for 30 min at 4 °C. Samples were cleared of cellular debris by centrifugation, and supernatants containing the nuclear fraction were subjected to immunoblot analysis using a mouse polyclonal antibody directed against the p65 (RelA) subunit of NF-kB (Millipore, Billerica, MA). Protein bands corresponding to NK-1R or RelA were detected using a Bio-Rad ChemiDoc imaging system, and quantification was performed using ImageLab software (Bio-Rad) normalized to the expression of the housekeeping gene product β-actin. NK-1R and p65 (RelA) protein expression is presented graphically as relative levels adjusted to β-actin expression, and the immunoblots shown are representative of at least three separate experiments.

### ELISA quantification of SP levels and IL-6 production

Levels of SP in ex vivo NHP cortical supernatants were determined using a commercially available ELISA kit according to the directions provided by the manufacturer (R&D Systems). IL-6 production by human glial cultures was assessed by specific capture ELISA using a rat anti-human IL-6 capture antibody and a biotinylated rat anti-human IL-6 detection antibody (BD Pharmingen). Bound antibody was detected by streptavidin-horseradish peroxidase (BD Biosciences) followed by the addition of tetramethylbenzidine (TMB) substrate. H_2_SO_4_ was used to stop the reaction, and absorbance was measured at 450 nm. Dilution of recombinant IL-6 (BD PharMingen) was used to generate a standard curve, and the IL-6 concentration in each supernatant was determined by extrapolation of absorbances to the standard curve.

### Fluorescent immunohistochemical analysis

Hμglia cells (1.5 × 10^5^) were plated on acid-washed glass coverslips coated with poly-d-lysine. Cells were fixed (2% PFA), permeabilized (with 50% acetone 50% methanol solution), and blocked (5% goat serum). Cells were stained with a monoclonal mouse antibody directed against NK-1R (clone ZN003, Thermo Scientific, Rockford, IL) and a polyclonal goat antibody directed against the microglial marker Iba1 (Abcam, Cambridge, MA) prior to incubation with secondary antibodies coupled to Alexa Fluor 488 or Alexa Fluor 594. Samples were mounted with Prolong Gold containing DAPI (Invitrogen) and imaged using an Olympus 1X71 inverted microscope and an Olympus DP70 digital camera.

### Flow cytometric analysis

U87-MG cells, primary human astrocytes, or hμglia cells, seeded in 12-well plates (1.5 × 10^5^) were unstimulated or exposed to bacterial products for 2 h prior to addition of an enzyme free dissociation buffer (ThermoFisher Scientific), washing, and blocking (5% normal goat serum). Cells were then stained with a monoclonal mouse antibody directed against NK-1R (clone ZN003, Thermo Scientific) followed by incubation with a secondary antibody coupled to either Alexa Fluor 488 or Alexa Fluor 594, prior to flow cytometric analysis using an Accuri C6 cytometer (BD Biosciences, Franklin Lakes, NJ).

### Assessment of soluble neurotoxic mediator production by infected human glia

Primary human astrocytes were uninfected or infected with *B. burgdorferi* or *S. pneumoniae* in the absence or presence of SP (5 nM). At 24 h following infection, conditioned medium was collected and placed on HCN-1A neuronal cells. At 24 h following addition of the conditioned medium, the viability of the HCN-1A cells was assessed by trypan blue exclusion in ten microscopy fields.

### Statistical analysis

Data are presented as the mean ± standard error of the mean (SEM). Statistical analyses were performed using Student’s two-tailed *t* test or a one-way analysis of variance (ANOVA) with Bonferroni’s or Tukey’s post hoc tests as appropriate using commercially available software (GraphPad Prism, GraphPad Software, La Jolla, CA). In all experiments, results were considered statistically significant when a *P* value of less than 0.05 was obtained.

## Results

### The full-length NK-1R isoform is constitutively and robustly expressed in the NHP brain, and SP levels are elevated following challenge with *B. burgdorferi*

**Fig. 1:**
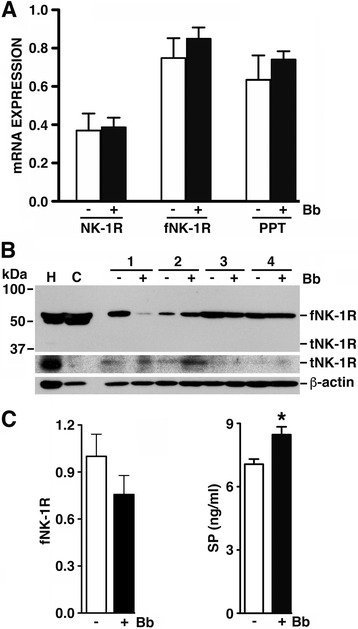
The full-length NK-1R isoform is expressed at robust levels in uninfected ex vivo rhesus macaque frontal cortical tissue, and SP levels are elevated in this tissue following *B. burgdorferi* infection. Cultured NHP brain tissue was uninfected (−) or infected (+) with *B. burgdorferi* (Bb, 1 × 10^7^ bacteria; *n* = 4). Panel **a** At 2 h following infection, tissue expression of mRNA encoding the combined isoforms of NK-1R (NK-1R), the full-length isoform of NK-1R (fNK-1R), and pre-pro-tachykinin (PPT), was determined by RT-PCR and relative expression normalized to GAPDH levels was determined by densitometric analysis. Panel **b** At 2 h, protein expression of fNK-1R, the truncated NK-1R isoform (tNK-1R), and the housekeeping gene product β-actin, was determined by immunoblot analysis for each of the four brain tissue samples (1 through 4) either constitutively or following ex vivo infection. Expression in HeLa human epithelial (H) and CATH.a mouse neuronal (C) cell lines is included as positive controls. With an extended imaging exposure time, low-level tNK-1R expression could be discerned in the representative blot shown (middle bands). Panel **c**: At 4 h, fNK-1R protein expression and SP levels were determined in infected and uninfected tissue samples (*n* = 4) by immunoblot analysis and normalized to β-actin expression and specific capture ELISA, respectively (Panel **c**). Data is expressed as the mean ± SEM and asterisk indicates a statistically significant difference from uninfected brain tissue (*p* < 0.05)

We have previously demonstrated the ability of an NK-1R antagonist to ameliorate CNS inflammation associated with in vivo CNS infection in a NHP model [10]. To begin to determine whether resident CNS cells, as distinct from infiltrating leukocytes, are responsive to SP, we have determined the constitutive expression of SP and NK-1R in rhesus macaque frontal cortical tissue and assessed the level of expression of these molecules following ex vivo bacterial challenge. As shown in Fig. 1a, NHP cortical tissue showed robust constitutive expression of mRNA encoding NK-1R, and the full-length NK-1R isoform (fNK-1R) in particular, in addition to pre-pro-tachykinin (PPT) mRNA that encodes SP. While levels of fNK-1R and PPT mRNA tended to increase at 2 h following exposure to *B. burgdorferi*, this effect was not statistically significant (Fig. 1a). Importantly, the expression of NK-1R mRNA was reflected in robust levels of fNK-1R, but not the truncated NK-1R isoform (tNK-1R), in uninfected brain tissue. Such expression was not significantly altered at 2 h (Fig. 1b) or 4 h (Fig. 1c) following infection. However, *B. burgdorferi* challenge did significantly elevate SP protein expression in NHP brain tissue above the high basal levels and within 4 h (Fig. 1c).

### Human microglia constitutively and functionally express NK-1R

**Fig. 2:**
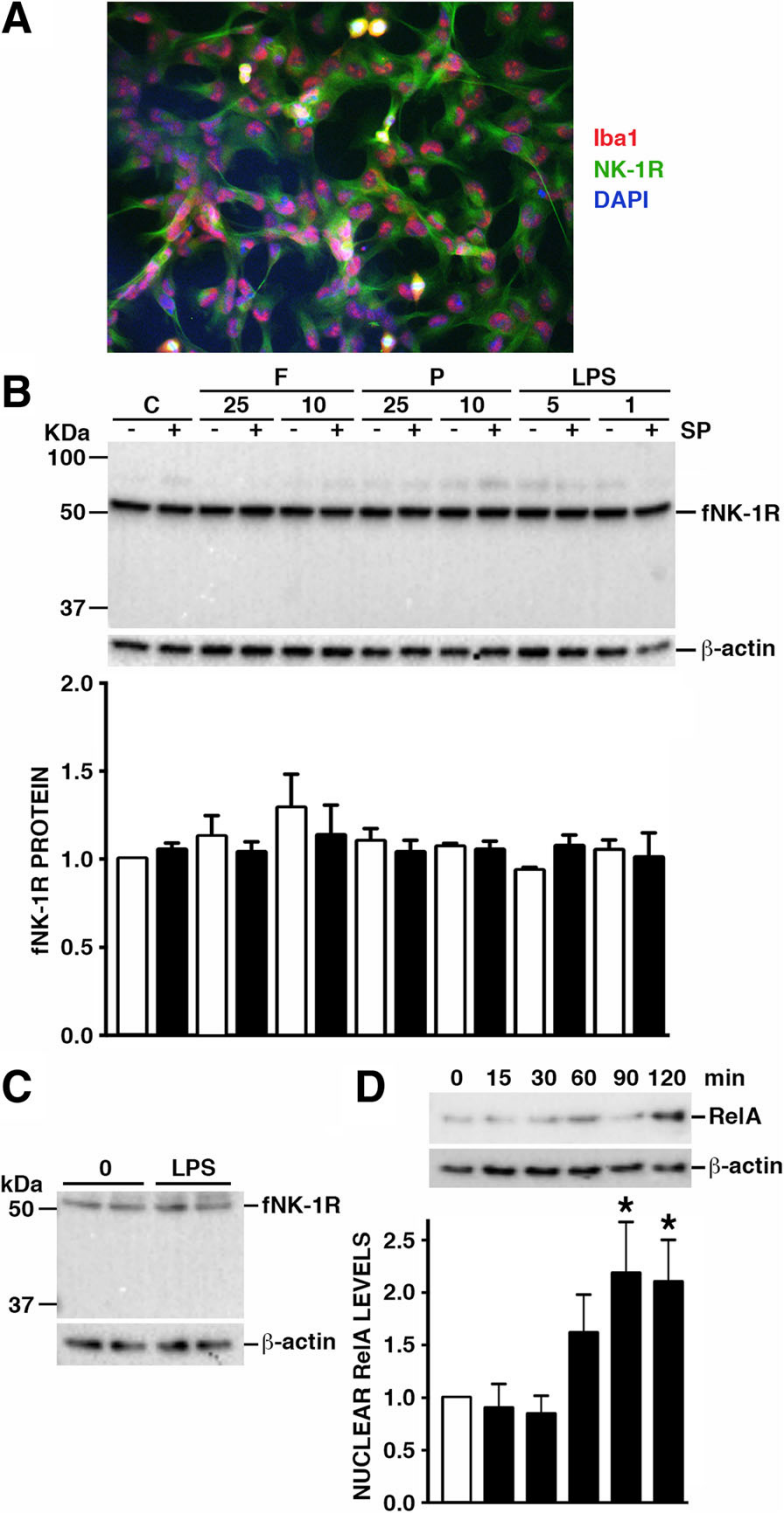
Human microglia constitutively and functionally express NK-1R. Panel **a** The hμglia immortalized human microglial cells are positive for Iba-1 (red) and constitutively express cell surface NK-1R (green) as determined by immunofluorescence microscopy. Nuclear staining is also shown in this representative image (DAPI: blue). Panel **b** hμglia cells were untreated (C) or exposed to bacterial flagellin (F: 10 or 25 ng/mL), Pam3Cys (P: 10 or 25 ng/mL), or LPS (1 or 5 ng/mL), in the presence or absence of SP (10 nM) for 18 h and protein expression of NK-1R and the housekeeping gene product β-actin was determined by immunoblot analysis (*n* = 4). The average relative expression of fNK-1R, determined by densitometric analysis and normalized to β-actin levels, is shown below the representative immunoblot. Panel **c** Primary human microglia were untreated (0) or exposed to LPS (5 ng/mL) for 18 h and protein expression of NK-1R and the housekeeping gene product β-actin were determined by immunoblot analysis (*n* = 2). Panel **d** Human microglia were untreated (0) or exposed to SP (10 nM) for 15, 30, 60, 90, and 120 min, and nuclear levels of NF-kB p65 (RelA) and the housekeeping gene product β-actin were determined by immunoblot analysis (*n* = 5). The average relative nuclear expression of RelA, determined by densitometric analysis and normalized to β-actin levels, is shown below the representative immunoblot. Data is expressed as the mean ± SEM and asterisks indicate a statistically significant difference from untreated cells (*p* < 0.05)

To begin to determine the ability of human glial cells to respond to SP, we have assessed the expression of NK-1R by microglia, the principal myeloid immune cell of the CNS. As shown in Fig. 2a, the human microglial cell line hμglia constitutively expresses NK-1R as determined by immunofluorescent microscopy. NK-1R expression by hμglia cells was confirmed by immunoblot analysis, which showed robust constitutive levels of fNK-1R protein in the absence of detectable tNK-1R isoform expression (Fig. 2b). Such robust fNK-1R expression was not elevated further following exposure to the TLR ligands, bacterial flagellin, PAM3Cys, and LPS, or exposure to whole intact *N. meningiditis*, *S. pneumoniae*, or *B. burgdorferi*, as determined by immunoblot analysis (Fig. 2b and data not shown) and flow cytometry (data not shown). Similarly, treatment with SP, alone or in combination with bacterial ligands, failed to significantly increase fNK-1R protein expression (Fig. 2b).

Importantly, we have extended these studies to primary human microglia and we show that they also constitutively express fNK-1R but not tNK-1R (Fig. 2c). Similar to hμglia cells, LPS challenge failed to elicit a significant effect on fNK-1R expression by primary human microglia (Fig. 2c).

In order to establish the functionality of NK-1R on human microglia, we assessed the ability of SP to elicit activation of NF-kB, a master regulator of inflammatory gene transcription. Consistent with our previous results in other myeloid immune cell types including macrophages and dendritic cells [16], SP induced nuclear translocation of the NF-kB p65 subunit (RelA) in human microglial cells (Fig. 2d) confirming the functional nature of NK-1R expression by this cell type.

### Human astrocytes constitutively express NK-1R, and bacterial challenge can elevate cell surface expression of this receptor by these cells

**Fig. 3:**
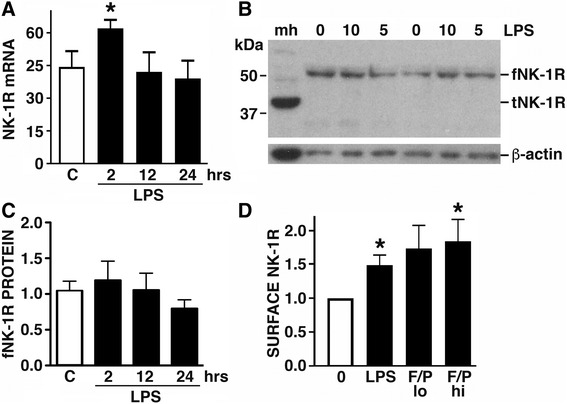
U87-MG astroglioma cells constitutively express NK-1R, and expression of this receptor is increased following exposure to bacterial components. Panel **a** U87-MG cells were untreated (C) for 24 h or exposed to LPS (5 ng/mL) for 2, 12, and 24 h, and the level of expression of mRNA encoding NK-1R was determined by RT-PCR (*n* = 4). Panel **b** Cells were untreated (0) or exposed to LPS (5 or 10 ng/mL) for 24 h and protein expression of fNK-1R, tNK-1R, and the housekeeping gene product β-actin, was determined by immunoblot analysis (*n* = 3). Expression in mouse heart tissue (mh) is included as a positive control for tNK-1R. Panel **c** Cells were untreated for 24 h (C) or exposed to LPS (5 ng/mL) for 2, 12, and 24 h, and the level of fNK-1R protein expression was determined by immunoblot analysis. Data is shown as protein levels, normalized to β-actin expression, relative to NK-1R expression in untreated cells (n = 4). Panel **d** Cells were untreated (0) or exposed to LPS (5 ng/mL) or bacterial flagellin plus PAM3Cys at 100 ng/mL and 500 ng/mL (lo) or 200 ng/mL and 1000 ng/mL (hi), respectively, and cell surface NK-1R expression was determined at 2 h by flow cytometry. Data is shown relative to cell surface NK-1R expression on untreated cells (*n* = 5). Data is expressed as the mean ± SEM and asterisks indicate statistically significant differences between untreated and treated cells (*p* < 0.05)

To begin to determine the ability of human astrocytes to respond to SP, we have assessed the expression of NK-1R by this cell type. As shown in Fig. 3a, the human astrocytic cell line U87-MG constitutively expresses mRNA encoding NK-1R, and these cells contain fNK-1R isoform protein in the absence of demonstrable tNK-1R expression (Fig. 3b, c). Activation of U87-MG cells with bacterial LPS elicited a transient increase in NK-1R mRNA levels at 2 h post-challenge (Fig. 3a), but this TLR4 ligand did not reproducibly elicit significant elevations in total cellular fNK-1R expression by these cells (Fig. 3b, c). Interestingly, LPS and a combination of the TLR2 and TLR5 ligands, Pam3Cys and bacterial flagellin, were able to significantly increase NK-1R expression on the surface of U87-MG cells as determined by flow cytometry (Fig. 3d).

**Fig. 4:**
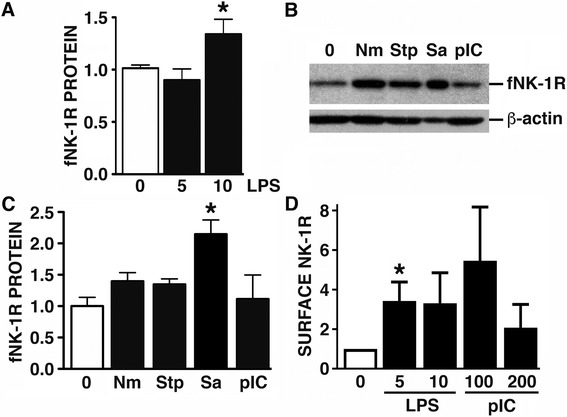
Cultured primary human astrocytes constitutively express NK-1R, and expression of this receptor is increased following bacterial challenge. Panel **a** Primary human astrocytes were untreated (0) or exposed to LPS (5 or 10 ng/mL) for 12 h, and the level of fNK-1R protein expression was determined by immunoblot analysis. Data is shown as protein levels, normalized to β-actin expression, relative to NK-1R expression in untreated cells. Panels **b** and **c** Cells were untreated (0) or exposed to *N. meningiditis* (Nm: MOI of 10:1 bacteria to human cells), *Streptococcus pneumoniae* (Stp: MOI 10:1), *Staphylococcus aureus* (Sa: MOI 100:1), or polyI:C (pIC), for 12 h, and the level of fNK-1R protein expression was determined by immunoblot analysis. A representative blot is shown and data is presented as protein levels, normalized to β-actin expression, relative to NK-1R expression in untreated cells (*n* = 4). Panel **d** Cells were untreated (0) or exposed to LPS (5 or 10 ng/mL) or polyI:C (100 or 200 ng/mL), and cell surface NK-1R expression was determined at 2 h by flow cytometry. Data is shown relative to cell surface NK-1R expression on untreated cells (*n* = 3). Data is expressed as the mean ± SEM and asterisks indicate statistically significant differences between untreated and treated cells (*p* < 0.05)

Importantly, we have extended these studies to primary human astrocytes and we show that they also constitutively express fNK-1R (Fig. 4a–c), but not tNK-1R (data not shown), and LPS treatment can similarly increase relative NK-1R mRNA expression levels (0.32 ± 0.03 versus 0.43 ± 0.05 in untreated and LPS treated cells, respectively; *p* < 0.05, *n* = 5) and cell surface NK-1R protein expression on these cells (Fig. 4d). In contrast to U87-MG cells, however, LPS challenge elicited modest but significant increases in the level of total cellular NK-1R protein levels in primary human astrocytes (Fig. 4a). Furthermore, exposure of these human glial cells to disparate bacterial pathogens tended to increase fNK-1R expression, and this effect was particularly marked in cells challenged with *S. aureus* (Fig. 4b, c). However, stimulation of primary human astrocytes with polyI:C, a double-stranded RNA mimetic and TLR3 ligand, yielded equivocal results with inconsistent effects on total fNK-1R protein expression (Fig. 4c) and cell surface NK-1R levels (Fig. 4d).

### SP augments the production of inflammatory and neurotoxic mediators by bacterially challenged human astrocytes

**Fig. 5:**
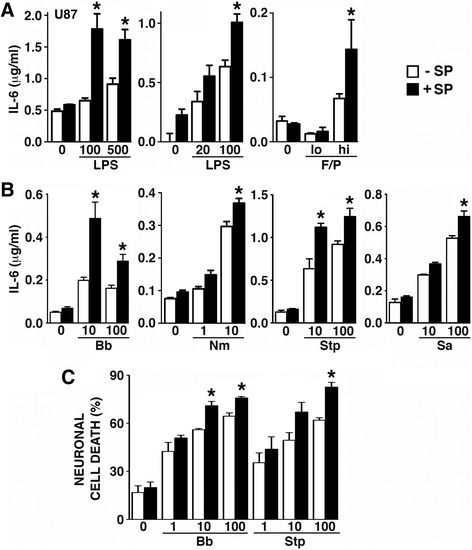
SP augments the production of inflammatory and neurotoxic mediators by bacterially challenged human astrocytes. Panel **a** U87-MG cells (labeled U87) or primary human astrocytes were untreated (0), or exposed to LPS (20–500 ng/mL) or bacterial flagellin plus Pam3Cys at 75 ng/mL and 500 ng/mL (lo) or 150 ng/mL and 1000 ng/mL (hi), respectively, in the absence (−SP) or presence (+SP) of recombinant SP (10 nM), for 24 h, and the level of IL-6 protein release was determined by specific capture ELISA (*n* = 3). Panel **b** Primary human astrocytes were untreated (0) or challenged with *B. burgdorferi* (MOI of 10 and 100:1 bacteria to human cells), *N. meningiditis* (Nm: MOI of 1 and 10:1), *S. pneumoniae* (Stp: MOI of 10 and 100:1), or *S. aureus* (Sa: MOI of 10 and 100:1), in the absence (−SP) or presence (+SP) of recombinant SP (5 nM), for 24 h and the level of IL-6 release was determined by specific capture ELISA (*n* = 3). Panel **c** Primary human astrocytes were untreated (0) or challenged with *B. burgdorferi* (MOI of 1, 10, and 100:1 bacteria to human cells) or *S. pneumoniae* (Stp: MOI of 1, 10, and 100:1), in the absence (−SP) or presence (+SP) of recombinant SP (5 nM) for 24 h. Conditioned medium from each was then placed on HCN neuronal cells and cell death was assessed by trypan blue exclusion at 24 h (*n* = 3). Data is expressed as the mean ± SEM and asterisks indicate statistically significant differences between the SP treated and untreated cells (*p* < 0.05)

To begin to determine the functional significance of NK-1R expression by human astrocytes, we have assessed the effect of SP on the production of the inflammatory mediator IL-6. As shown in Fig. 5a, SP failed to elicit significant IL-6 production by either U87-MG astrocytic cells or primary human astrocytes. However, SP significantly augmented the production of this cytokine by both U87-MG cells and primary astrocytes stimulated by bacterial LPS or a combination of bacterial flagellin and Pam3Cys (Fig. 5a and data not shown).

In addition to these TLR agonists, we have assessed whether SP can augment the immune responses of human astrocytes to disparate and clinically relevant bacterial pathogens of the CNS. As shown in Fig. 5b, SP significantly increased IL-6 secretion by primary human astrocytes in response to the Gram-negative bacteria *B. burgdorferi* and *N. meningiditis*, and the Gram-positive organisms *S. pneumoniae* and *S. aureus*. Furthermore, we determined that SP can enhance the production of soluble mediators capable of inducing neuronal cell death by human astrocytes in response to either *B. burgdorferi* or *S. pneumoniae* (Fig. 5c).

## Discussion

Bacterial infections of the CNS constitute a group of highly damaging and often life-threatening diseases. What makes the etiology of these diseases so perplexing is that severe CNS inflammation can be initiated by bacterial species that are generally regarded to be of low virulence [17]. While such responses may be protective, inflammation elicited by infectious agents often results in progressive CNS damage. Indeed, we have recently demonstrated that inflammation plays a key role in pathogenesis in a NHP model of acute Lyme neuroborreliosis [18]. A hallmark of developing inflammation is the synergistic interaction between cells and their products that can amplify the response. It is now widely accepted that SP, the most abundant tachykinin in the CNS, can exacerbate the inflammatory responses of both leukocytes and resident glial cells via the high affinity full-length NK-1R isoform (as reviewed in [1, 2]). Importantly, we have demonstrated that SP can augment proinflammatory mediator production by murine glia in response to bacterial challenge [9]. Consistent with this finding, we have reported that endogenous SP/NK-1R interactions are required for maximal proinflammatory cytokine expression in vivo following direct CNS administration of *N. meningitidis* or *B. burgdorferi* in mice [9]. In addition, we have shown that an NK-1R antagonist can attenuate the neuronal and glial production of inflammatory mediators including CCL2 and IL-6 in rhesus macaque frontal cortex explants and isolated DRG cells following *B. burgdorferi* challenge [19]. Furthermore, we have recently demonstrated that NK-1R antagonist treatment can attenuate aspects of the bacteria-induced inflammatory responses in CNS tissues in an in vivo NHP model of Lyme neuroborreliosis [10].

In the present study, we have confirmed the robust expression of fNK-1R in NHP cortical brain tissue, with negligible expression of the truncated low affinity isoform (as described in [20]) that has been reported to lack the ability to elicit proinflammatory responses in other cell types [21, 22]. In contrast to our studies in the NHP brain cortex at 2 weeks following in vivo *B. burgdorferi* infection [10] and a report in the rat spine following chronic stress [23], we have shown that acute ex vivo challenge with *B. burgdorferi* fails to elicit significant changes in NK-1R mRNA or protein expression above constitutive levels. However, *B. burgdorferi* infection did elicit a statistically significant elevation in SP protein levels within brain tissue, indicating that the expression of neurokinin signaling components can be modulated in situ in response to bacterial challenge.

We have previously documented the functional expression of NK-1R by peripheral myeloid immune cell types including macrophages and dendritic cells [24, 25]. However, the expression of the SP receptor by microglia has been more contentious. Early findings indicated the absence of NK-1R expression by rat microglia based upon SP binding studies [26] while another group reported the lack of NK-1R expression by activated rat microglia following cerebral ischemia [27]. In contrast, one study reported the presence of NK-1R in human fetal microglia [28] and we have previously shown the functional expression of NK-1R by primary murine microglia [11]. In the present study, we have demonstrated the constitutive expression of full-length NK-1R protein by both a human microglial cell line and primary human microglia as determined by immunoblot analysis, immunohistochemical staining, and flow cytometry, at robust levels that could not be further elevated by exposure to bacterial ligands for TLR2, TLR4, or TLR5, either alone or in combination with SP treatment. In agreement with our results in ex vivo NHP cortical brain tissue, we were not able to detect significant levels of the truncated NK-1R isoform in human microglial cells. Furthermore, we have proved that fNK-1R is functionally expressed by human microglial cells with the demonstration that SP can elicit the activation of the critical proinflammatory transcription factor NF-kB, which is consistent with our prior studies in murine macrophages, dendritic cells, and microglia [11, 16].

In contrast to microglia, the expression of NK-1R by astrocytes has been more clearly established with the demonstration of this receptor in primary cortical mouse and rat astrocytes [29–31]. Furthermore, human brain astrocytes have been reported to express NK-1R, albeit at markedly lower levels than that seen in spinal cord cells [32]. However, it should be noted that the NK-1R isoform expressed was not defined in these studies, and at least one group has failed to detect the presence of this receptor in activated rat astrocytes following an ischemic insult [27]. Here, we show that both U87-MG human astrocytic cells and primary human cortical astrocytes express NK-1R mRNA and the full-length isoform protein as determined by immunoblot analysis and flow cytometry. Interestingly, we have found that exposure to bacterial components that serve as ligands for TLRs can elevate NK-1R mRNA expression and cell surface protein expression by U87-MG cells. Furthermore, challenge with bacteria or their products can elevate total cellular and cell surface NK-1R protein levels by primary human astrocytes. An elevation in NK-1R expression by astrocytes following exposure to activating stimuli is consistent with the documented ability of inflammatory mediators to increase NK-1R levels in U87-MG cells and primary rat astrocytes [33] and leukocytes [24, 34].

In accord with previous studies in human spinal astrocytes and primary rat astrocytes [26, 32], SP failed to induce significant IL-6 production by either U87-MG cells or primary human astrocytes when used as the sole stimulus. However, SP significantly augmented cytokine responses by both cell types following exposure to bacterial TLR ligands. This finding is in agreement with the work of Luber-Narod and colleagues [26] in rat astrocytes, but contrasts with another early report that SP does not affect the responses of human cortical astrocytes [32]. Importantly, we have shown that SP can significantly elevate the production of IL-6 or soluble neurotoxic mediators induced by disparate Gram-negative and Gram-positive bacterial pathogens of the CNS, including *B. burgdorferi*, *N. meningitidis*, *S. pneumoniae* and, to a lesser extent, *S. aureus*.

Taken together, the robust constitutive and functional expression of the full-length NK-1R isoform by human microglia and astrocytes, and the ability of SP to augment inflammatory signaling pathways and mediator production by these cells, support the contention that SP/NK-1R interactions play a significant role in the damaging neuroinflammation and neurological sequelae associated with bacterial infections of the CNS in human subjects. Furthermore, given the available data that SP/NK-1R interactions also augment detrimental inflammation during parasitic CNS infections and perhaps multiple sclerosis, while contributing to neuroprotection during some degenerative CNS disorders and intracellular viral/bacterial infections (as discussed in [2]), the functional expression of NK-1R by human glial cells may have broader implications. Clearly, further investigation of the ability of SP to augment CNS inflammation following infection and the benefits of targeting NK-1R in such clinical conditions is warranted.

## Conclusions

Our results show that the NHP brain as well as human microglial and astrocytic cells constitutively express robust levels of the full-length isoform of the high affinity SP receptor, NK-1R. In addition, we demonstrate that the expression of NK-1R by human astrocytes can be further elevated following exposure to disparate bacterial pathogens or their components. Importantly, we have demonstrated that NK-1R is functional in both human microglia and astrocytes and show that SP can augment the inflammatory immune responses of both CNS cell types. Such an effect may underlie the previously documented ability of an NK-1R antagonist to attenuate inflammation in a NHP model of CNS infection.

## Abbreviations

ANOVA: Analysis of variance
cDNA: Complementary deoxyribonucleic acid
CNS: Central nervous system
DAPI: 4′,6-Diamidino-2-phenylindole
DNA: Deoxyribonucleic acid
ELISA: Enzyme linked immunosorbent assay
FBS: Fetal bovine serum
fNK-1R: Full-length neurokinin-1 receptor isoform
GAPDH: Glyceraldehyde 3-phosphate dehydrogenase
IL: Interleukin
LB: Lysogeny broth
LPS: Lipopolysaccharide
MOI: Multiplicity of infection
mRNA: Messenger ribonucleic acid
NF-kB: Nuclear factor kappa-light-chain-enhancer of activated B cells
NHP: Nonhuman primate
NK-1R: Neurokinin-1 receptor
PFA: Paraformaldehyde
poly I:C: Polyinosinic:polycytidylic acid
PPT: Preprotachykinin
RNA: Ribonucleic acid
RT-PCR: Reverse-transcribed polymerase chain reaction
SEM: Standard error of the mean
SP: Substance P
TLR: Toll-like receptor
tNK-1R: Truncated neurokinin-1 receptor isoform
